# Comparison of biological and mechanical properties of different paranasal sinus mucosa in goat

**DOI:** 10.1186/s12903-022-02233-y

**Published:** 2022-05-25

**Authors:** Xinyu Fang, Cai Mi, Yingdi Wang, Yidan Sun, Jia Nie, Haoning Tang, Yajing Liu, Yanfeng Li, Jing Wang, Zheng Ma, Yishi Han

**Affiliations:** 1 Postgraduate Training Base, Jinzhou Medical University and The Fourth Medical Center, Chinese PLA General Hospital, Beijing, China; 2 Department of Stomatology, the Fourth Medical Center, Chinese PLA General Hospital, No.51 Fucheng Road, Haidian District, Beijing, 100048 China; 3 Department of Stomatology, AMHT Group Aerospace 731 Hospital, Beijing, China

**Keywords:** Maxillary sinus augmentation, Sinus mucosa, Mucosal thickness, Maximum endurable pressure intensity

## Abstract

**Objective:**

The present study was designed to explore endurable pressure intensity of different paranasal sinus mucosa in goats.

**Method:**

Mucosa commonly involved in maxillary sinus augmentation, including mucosa from maxillary sinus crest, maxillary sinus floor, and frontal sinus, were harvested in a computed tomography-guided manner. The obtained mucosa was then sectioned into square and irregular ones for maximum endurable pressure intensity determination and morphological observation, respectively.

**Results:**

Thickness of paranasal sinus mucosa, as determined under morphological staining by an optical microscope with a graduated eyepiece, were calculated. And the results showed that the average thickness of maxillary sinus crest mucosa, floor mucosa, and frontal sinus mucosa in goats were 410.03 ± 65.97 μm, 461.33 ± 91.37 μm and 216.90 ± 46.47 μm, respectively. Significant differences between maxillary sinus crest and frontal sinus, maxillary sinus floor, and frontal sinus were observed (*P* < 0.05). Maximum endurable pressure intensity was determined by utilizing a self-made clamp device and the results revealed maximum endurable pressure intensity of maxillary sinus crest mucosa, floor mucosa and frontal sinus mucosa in goats were 260.08 ± 80.12Kpa, 306.90 ± 94.37Kpa and 121.72 ± 31.72Kpa, respectively. Also, a statistically significant difference was observed when comparing the endurable pressure intensity between maxillary sinus crest and frontal sinus, maxillary sinus floor, and frontal sinus (*P* < 0.05). Further correlation analysis also revealed a positive correlation between the thickness of mucosa of the maxillary sinus and frontal sinus and maximum endurable pressure intensity (*P* < 0.05).

**Conclusion:**

Mucosal thickness and maximum endurable pressure intensity of maxillary sinus crest and floor were larger than that of frontal sinus mucosa and a positive correlation between the thickness of mucosa and endurable pressure intensity was observed. Our results thus might provide an experimental basis and guidance for mucosa-related problems involved maxillary sinus augmentation.

**Supplementary Information:**

The online version contains supplementary material available at 10.1186/s12903-022-02233-y.

## Introduction

Edentulism, as previously described as “the final marker of disease burden for oral health”[1], is quite a devastating and irreversible condition [2]. The prevalence of complete edentulism among older adults (> 50 years) in China [3] was about 9% and 4.9% in the United States [4] among adults over 15 years of age and above. A more recent study revealed that the prevalence of edentulism in Indonesia [5] among 80 years and older individuals is 29.8% with an overall prevalence of 7.8%. Teeth loss could affect mastication, speech and may result in poor esthetics, which greatly affects the quality of life of patients. Clinically, prosthetic implants were one of the most effective treatments for patients with edentulism. It is well known that success in implant dentistry depends on several parameters that may improve considering both biologic and mechanical criteria. One of the crucial factors is the osseointegration surrounding the implant, which requires the bone tissue to be sufficiently thick [6]. Limited by alveolar atrophy and insufficient residual bone height, implant under the condition of maxillary posterior tooth loss is difficult to perform. In those cases, a method of maxillary sinus augmentation (MSA), aimed to elevates the position of maxillary sinus floor and improves the fixation and stability of the implant in the maxilla were invented for treating maxillary posterior tooth loss. MSA was performed by lifting the maxillary sinus mucosa so that bone or bone substitute [7] could be implanted between the bone wall and the sinus mucosa of maxillary sinus floor. And the status of maxillary sinus mucosa after MSA could critically affect the effect of implantation. Therefore, explorations of biological and mechanical properties of different paranasal sinus mucosa were of great clinical significance while little research regarding this content was seen.

The paranasal sinuses are air-filled, bony cavities that lie within the facial bones of the skull, adjacent to the nasal passages. Humans have four paired paranasal sinuses, frontal, maxillary, sphenoid, and ethmoid, all extending from the respiratory area of the nasal cavity, and named after the bones they are found in [8]. The maxillary sinuses are the largest of all paranasal sinuses and are located bilaterally within the maxilla bone, assuming a pyramidal shape [9]. However, although significant efforts have been made to improve the techniques and design novel tools for crestal maxillary sinus elevation [10], mmaxillary sinus membrane perforation is the most common complication for maxillary sinus floor elevation [11]. Therefore, the mechanical properties are crucial for MSA.The anatomical structure of the maxillary sinus of goats, including location, size, shape, and bone structure, is very similar to that of humans. The width of the lateral sinus floor gradually widens from front to back. The lateral parietal bone plate is thinner, making it easier to drill, open a window, separate, and lift [12, 13]. The anatomical thickness and organization of the sinus mucosa of goats are also similar to those of humans [14].

Therefore, we utilized goat, whose paranasal anatomic structure is closely like human, to investigate biological and mechanical properties of different paranasal sinus mucosa for identifying the thickness and maximum endurable pressure intensity of different paranasal sinus mucosa. And thus might provide experimental mechanical properties of mucosal characteristics for MSA.

## Materials and methods

### Animals

The experimental and operation procedures of the current study were approved by Chinese PLA General Hospital. Six goats, aged 1 to 2 years old, were obtained from Animal Experimental Center of the first affiliated Hospital of the Chinese people's Liberation Army General Hospital. All goats involved were in similar body size and in good health without abnormality in oral cavity and maxillary sinus.

## Paranasal sinus mucosa sampling

Sampling of sinus mucosa was performed referring to a part of the previous study [15]. Generally, goats were anesthetized and then sacrificed. The goats were euthanized by inhalation of excess CO2. Procedures involving animals and their care were conducted in conformity with NIH guidelines (NIH Pub. No. 85–23, revised 1996) and were approved by Animal Care and Use Committee of Chinese PLA General Hospital. Our study covers the 3Rs (refinement, replacement, and reduction) and also outlines the procedures dealing with humane endpoints and pain management. The harvested head of goats was then subjected to computed tomography. Three-dimensional reconstruction and analysis were performed immediately after the scanning. Then the head was sectioned to obtain each side of the maxillary sinus according to the reconstruction results and its anatomical properties. Briefly, osteotomy lines were drawn in the maxillofacial region of the goat as guided by the CT imaging (Additional file 1: Fig. S1). Each maxillary sinus was divided into two parts: top and bottom. After the jaw was sawed, the top, bottom, and frontal sinus of the maxillary sinus were obtained (Additional file 2: Fig. S2). Mucosal of different locations were then genteelly separated. Each mucosa was sectioned to obtain a 20 × 20 mm square mucosa and an irregular mucosa. The square mucosa was immersed in normal saline containers. The irregular mucosa was placed in a 10% neutral formalin specimen bottle. The mucosa was fixed for 24 h and sent to the Department of Pathology for further examination.

## Thickness detection by morphological observation

Fixed mucosa was embedded in paraffin, sectioned, rehydrated and subjected to hematoxylin/eosin staining. The slides were sealed with neutral gum and observed under an optical microscope with a calibration eyepiece. The layered part of the mucous membrane was selected as the measurement area.

## Detection of maximum endurable pressure intensity

**Fig. 1:**
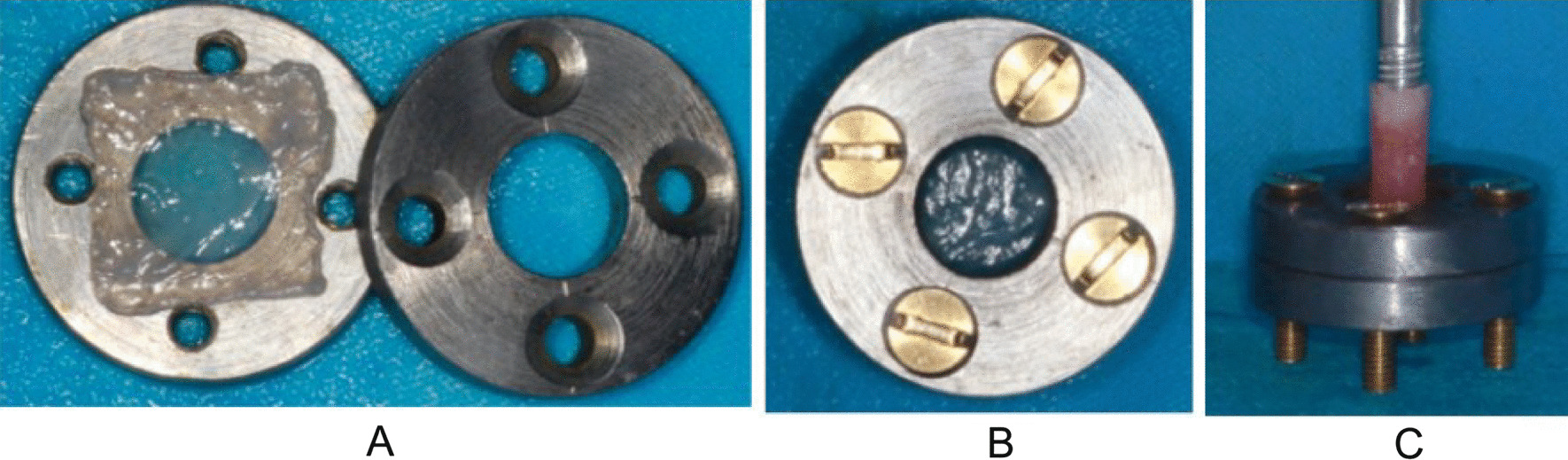
Self-made clamp device. **A** harvested square mucosa were placed between two self-made clamps. **B** the self-made clamp was carefully sealed with the screw. **C** the vertical downward pressure was applied evenly to the central mucosa exposed by the inner ring of the clamp. A force gauge was placed on the thrust end to record the pressure given

The 20 × 20 mm square mucosa were placed on a self-made clamp device (as presented in Fig. 1). Then the device was sealed, and the manometer was directly connected to the force section. During the process of pressure given, the mucous membrane gradually narrowed and became thinner and transparent. Pressure values were counted once mucosa was ruptured.

## Statistical analysis

SPSS21.0 was used for statistical analysis in this study. Data were presented as mean ± SD. One-way analysis of variance (ANOVA) was used for comparison of means among three groups, and the least significant difference (LSD) method was used for post-hoc tests. Pearson correlation analysis was performed to evaluate the relationship between mucosa thickness and pressure. A *P* value less than 0.05 was considered statistically significant.

## Results

### Comparison of mucosal thickness among the three groups

**Fig. 2:**
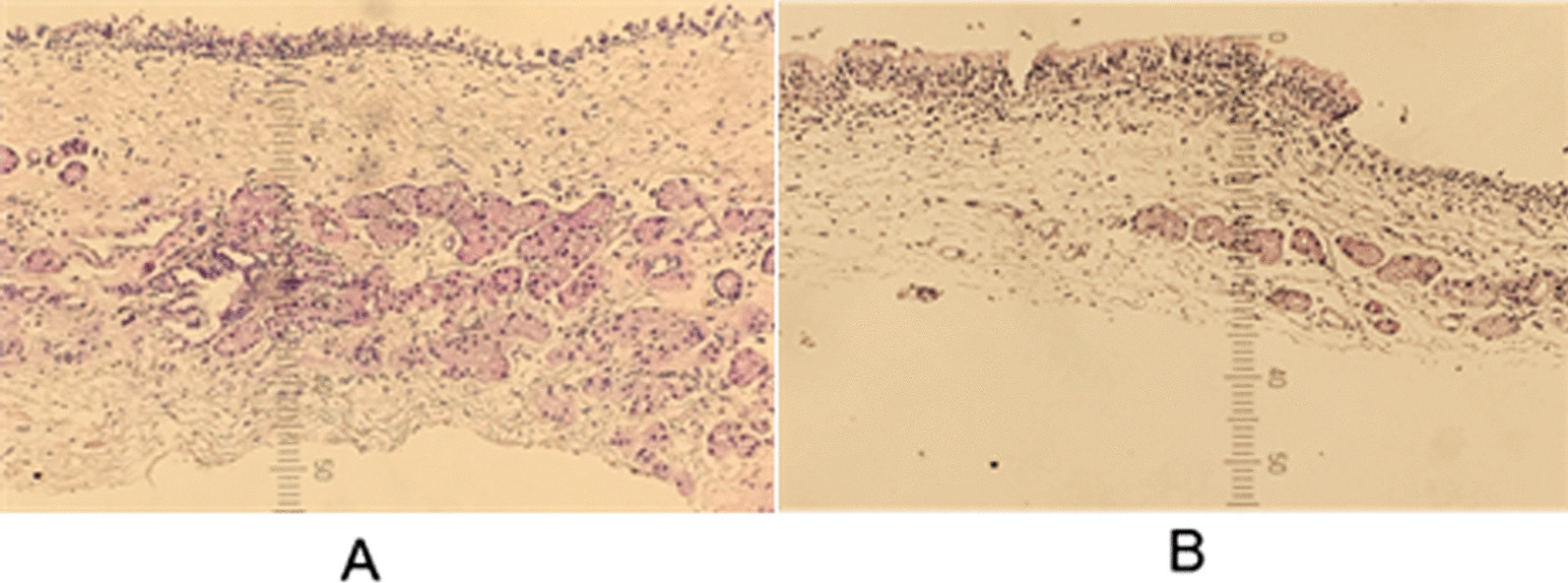
Representative image of hematoxylin/eosin staining of the floor of the maxillary sinus **A** and frontal sinus **B**

The thickness of mucosa at the crest and floor of maxillary sinus, and frontal sinus were measured under the microscope (Fig. 2). Results showed that the mucosa of the goat at the crest and floor of maxillary sinus was significantly thicker than that at the frontal sinus (One-way ANOVA, *P* < 0.001; LSD post-hoc test, maxillary sinus crest, and frontal sinus, *P* < 0.001; maxillary sinus floor and frontal sinus, *P* < 0.001, Table 1), but there was no significant difference between crest and floor of maxillary sinus.

**Table 1:** Mucosal thickness among the three groups

	Crest of maxillary sinus (*N* = 12)	Floor of maxillary sinus (*N* = 12)	Frontal sinus (*N* = 12)	*P*
Thickness (μm)	410.03 ± 65.97^*^	461.33 ± 91.37^*^	216.90 ± 46.47	< 0.001

^*^ Compared with frontal sinus, *P* < 0.001

## Comparison of maximum endurable pressure intensity among three groups

The maximum endurable pressure intensity was the pressure when mucosa was ruptured. Our results showed that the crest and floor of maxillary sinus had a significantly larger tensile strength than that of the frontal sinus (One-way ANOVA, *P* < 0.001; LSD post-hoc test, maxillary sinus crest and frontal sinus, *P* < 0.001; maxillary sinus floor and frontal sinus, *P* < 0.001, Table 2), but the difference between crest and floor of maxillary sinus were not statistically significant.

**Table 2:** Comparison of maximum endurable pressure intensity among three groups

	Crest of maxillary sinus (*N* = 12)	Floor of maxillary sinus (*N* = 12)	Frontal sinus (*N* = 12)	*P*
Pressure (Kpa)	260.08 ± 80.12^*^	306.90 ± 94.37^*^	121.72 ± 31.72	< 0.001

^*^ Compared with frontal sinus, *P* < 0.001

## Correlation between mucosal maximum endurable pressure intensity and thickness

**Fig. 3:**
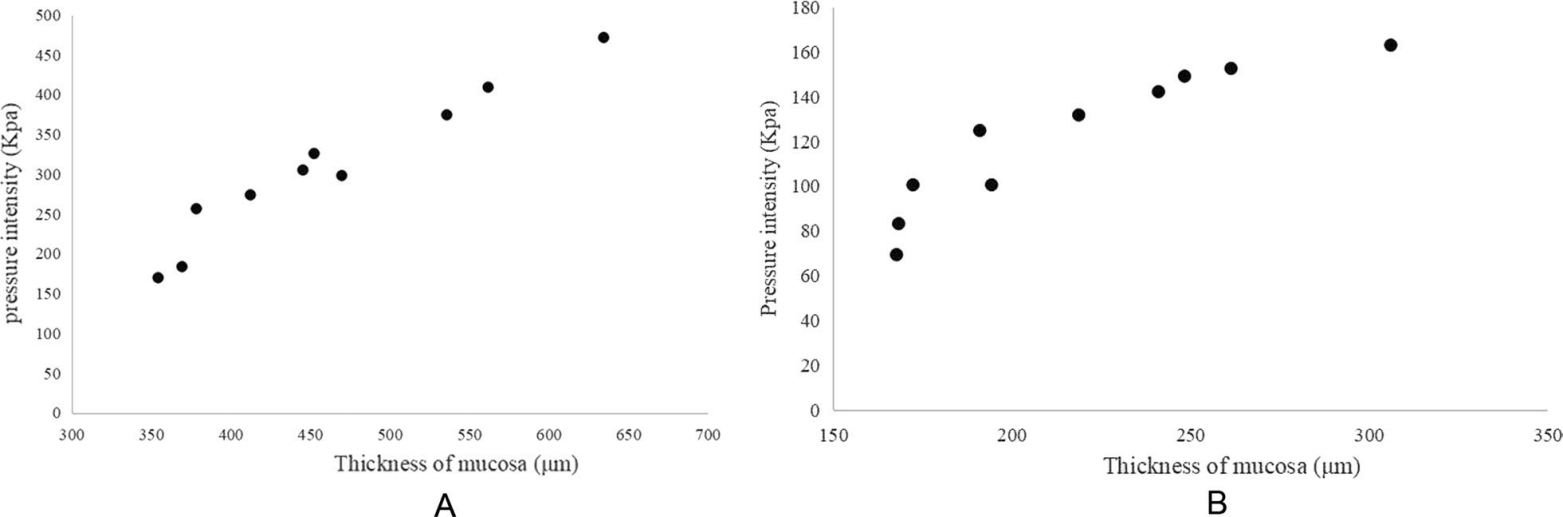
**A** Linear correlation regression equation between the maximum endurable pressure intensity (dependent variable) and thickness (independent variable) for maxillary sinus. **B** Linear correlation regression equation between the maximum endurable pressure intensity (dependent variable) and thickness (independent variable) for frontal sinus

To further investigate the relationship between mucosal thickness and maximum endurable pressure intensity, correlation analysis was performed. Since there were no significant differences in the thickness and tensile strength between the crest and floor of maxillary sinus, data of the two groups were pooled for analysis. The results showed that the tensile strength was positively correlated with the thickness of mucosa at the maxillary sinus (*r* = 0.976, *P* < 0.001, Table 3). Similarly, the tensile strength of the frontal sinus mucosa was positively related to the thickness (*r* = 0.920, *P* < 0.001, Table 3). In addition, a linear correlation regression equation between the tensile strength (dependent variable) and thickness (independent variable) was established for maxillary sinus and frontal sinus, respectively. Results showed that the regression equation was y = −176 + 1.055x (*P* < 0.001, Fig. 3A) for maxillary sinus, and y = -14.520 + 0.628x (*P* = 0.029, Fig. 3B) for frontal sinus, respectively.

**Table 3:** Correlation between maximum endurable pressure intensity and thickness of mucosa

Paranasal sinus	*r*	*R*.^2^	*P*
Maxillary sinus	0.976	0.953	< 0.001
Frontal sinus	0.920	0.847	< 0.001

## Discussion

The current study had preliminarily revealed the biological and mechanical properties of different paranasal sinus mucosa in goats. According to the results, we have found that thickness and mechanical properties of different paranasal sinus mucosa varies differently with mucosa of maxillary sinus crest and floor are significantly thicker and had larger tensile strength than that of frontal sinus. Further linear correlation analyzes revealed that the maximum endurable pressure intensity was positively related to thickness of mucosa at the maxillary sinus and frontal sinus in goats. Our research had provided beneficial experimental data for improving effects of maxillary sinus augmentation.

Maxillary sinus, composed of lateral wall of the nasal cavity with the shape of triangular cone, is located in the maxilla and is the largest cavity of the four paranasal sinuses. The average volume of maxillary sinus in the adult is 13 ml (Ranges from 12-15 ml) [16]. Various uneven crests, which are unrelated to age and sex and vary in individuals, were presented in the maxillary sinus and were thought to increases the risk of mucosal perforation and the risk of maxillary sinus augmentation [12, 17]. Researches revealed that average thickness of human maxillary sinus mucosa was about 0.3 ~ 0.8 mm with a color of blue and property of elasticity in healthy state [18]. Composed of pseudostratified ciliated columnar epithelium, lamina propria, and periosteal layer, mucosa of human maxillary sinus floor were proved to have the ability to promote osteogenesis [19] since maxillary sinus mucosal cells can be induced to express cytokines such as alkaline phosphatase, bone morphogenetic protein 2, osteopontin, mucin, and osteocalcin. Therefore, utilizing human maxillary sinus floor was a helpful direction for successful implantation.

As for the maximum endurable pressure intensity, the paired comparison of the average maximum endurable pressure intensity of mucosa in different positions of paranasal sinuses in goats showed that there were differences between the frontal sinus and the crest of the maxillary sinus and the floor of the maxillary sinus, but there was no statistical difference between the top and bottom of the maxillary sinus. The average maximum endurable pressure intensity of the mucosa at the crest and floor of the maxillary sinus was larger than that of the frontal sinus, and the floor of the maxillary sinus was larger than that at the crest of the maxillary sinus, with a minimum value of 69.36Kpa in the frontal sinus and a maximum value of 471.62Kpa in the floor of the maxillary sinus. The average maximum endurable pressure intensity of maxillary sinus crest, maxillary sinus floor, and frontal sinus mucosa was significantly different, which was also considered to result from the variation rate of maxillary sinus crest, sinus floor, and frontal sinus mucosa. There was a positive correlation between the maximum endurable pressure intensity and the thickness of the maxillary sinus and frontal sinus mucosa, but the correlation in frontal sinus mucosa was not strong. The determination coefficient of tensile strength-thickness of maxillary sinus mucosa was *R*^2^ = 0.953, which indicated that 95.3% of the difference in maximum endurable pressure intensity of maxillary sinus mucosa was caused by the thickness of maxillary sinus mucosa, that was, the difference in mucosal thickness can explain 95.3% of the difference in tensile strength. The determination coefficient of tensile strength-thickness of frontal sinus mucosa was *R*2 = 0.847, indicating that 84.7% of the differences in tensile strength of frontal sinus mucosa were caused by mucosal thickness. It indicated that the maximum endurable pressure intensity can be calculated from the thickness of maxillary sinus mucosa, which provided a reference basis for related biological characteristics in maxillary sinus lifting.

Mucosal mechanical properties, mucosal thickness, mucosal cell biology are the leading research topic regarding maxillary sinus augmentation. Currently, few experiments on the thickness and pressure tolerance of maxillary sinus mucosa were reported as in the researches related to mucosal mechanical properties [20]. Previous research mainly focused on single-factor, static and discrete observation, and analysis, which lacks horizontal and vertical comparison and cannot fully obtain the view of stress distribution information of mucous membrane under pressure. As for mucosal thickness-related research, mucosa paraffin-embedded slices were commonly utilized. Of particular, it should be ensured that the slices were completely perpendicular to the mucosa and appropriate fixation buffer and stain method were applied. Further researches were encouraged to develop methodology in measuring the thickness of mucosal more accurately. Last but not least, since the mucosal cells were proved to promote osteogenesis and to support the structure of maxillary sinus [19], it is also an important area of study the behavior of mucosal cells during maxillary sinus augmentation. Due to the complex structure and mechanical properties of human biological tissue, such as irregular maxillary structure, numerous and heterogeneous cavities, and individual differences, the effects of the angle and the shape of implant (cylindrical, conical, threaded, non-threaded) on the outcome of maxillary sinus lifting needed to be considered, so further clinical experiments and studies on mucosal characteristics should be conducted in the future.

There are some limitations of this study. Firstly, the sample size of the current study is relatively small, and we only used goats as our experimental subject. Further study should include more sample size and more kinds of animals. Secondly, the mechanical properties involved in the current study only included the maximum endurable pressure intensity, other mechanical properties related to maxillary sinus augmentation should also be included in further study. Thirdly, we did not investigate the cellular differences between sinus mucosa in this study, which should be further explored. Finally, this was.

an in-animal experiment, the human-derived tissues and cells will also be used in future experiments.

## Conclusion

In conclusion, this study presented that thickness and mechanical properties of different paranasal sinus mucosa varies differently: mucosa of maxillary sinus crest and floor is significantly thicker and had larger tensile strength than that of the frontal sinus. And maximum endurable pressure intensity was positively related to the thickness of mucosa at the maxillary sinus and frontal sinus in goats. Therefore, frontal sinus might not be helpful for providing experimental mechanical properties of mucosal characteristics for MSA. Our research had might help guide maxillary sinus augmentation operation.

## Supplementary Information

**Additional file1: Fig.S1** The osteotomy lines were drawn in the maxillofacial region of the goat as guided by the CT imaging.

**Additional file2:** **Fig.S2** A schematic drawing for maxillary sinus (the picture above, left) and frontal sinus (the picture above, right). A sagittal plane for maxillary sinus (the picture below).

## Data Availability

The datasets generated and analyzed during the current study are available from the corresponding author on reasonable request.
