# Supplementary figures and images for: Comparison of biological and mechanical properties of different paranasal sinus mucosa in goat
Source: BMC Oral Health. 2022 May 25;22:203. doi: 10.1186/s12903-022-02233-y (PMC9131649; doi:10.1186/s12903-022-02233-y)

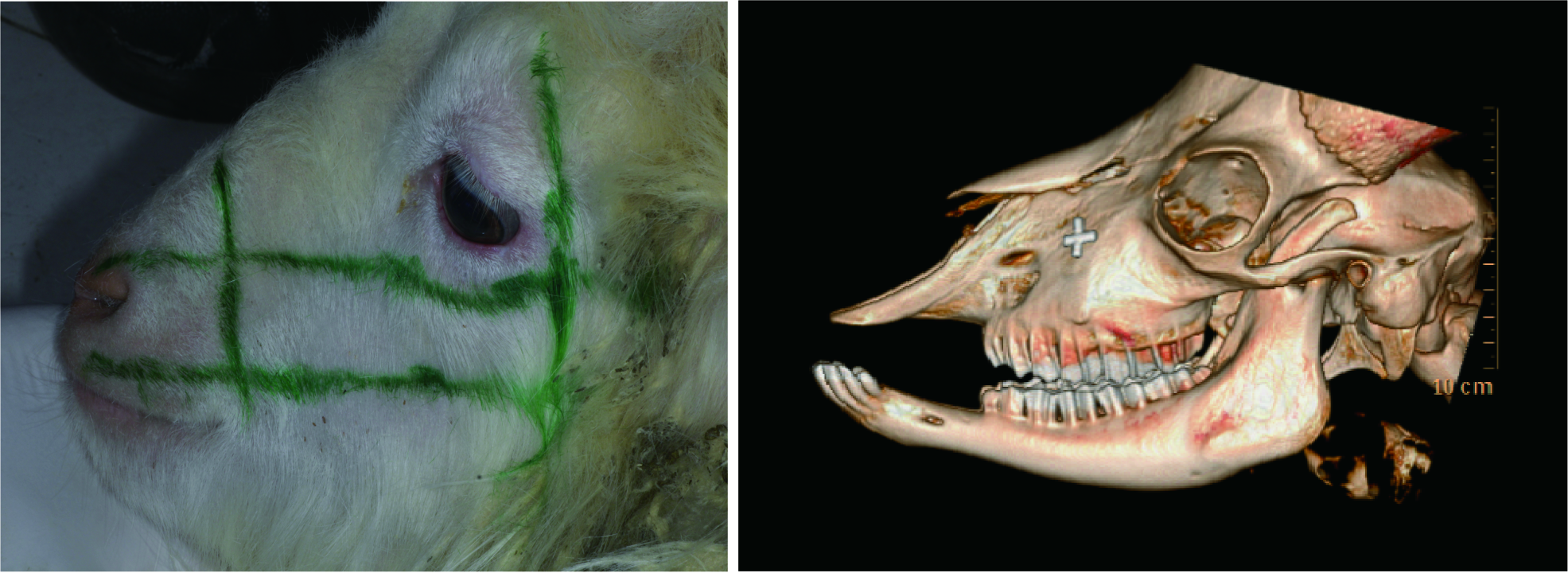

Supplement: Supplementary file 1 — Additional file1: Fig.S1 The osteotomy lines were drawn in the maxillofacial region of the goat as guided by the CT imaging. [file 12903_2022_2233_MOESM1_ESM.tif]

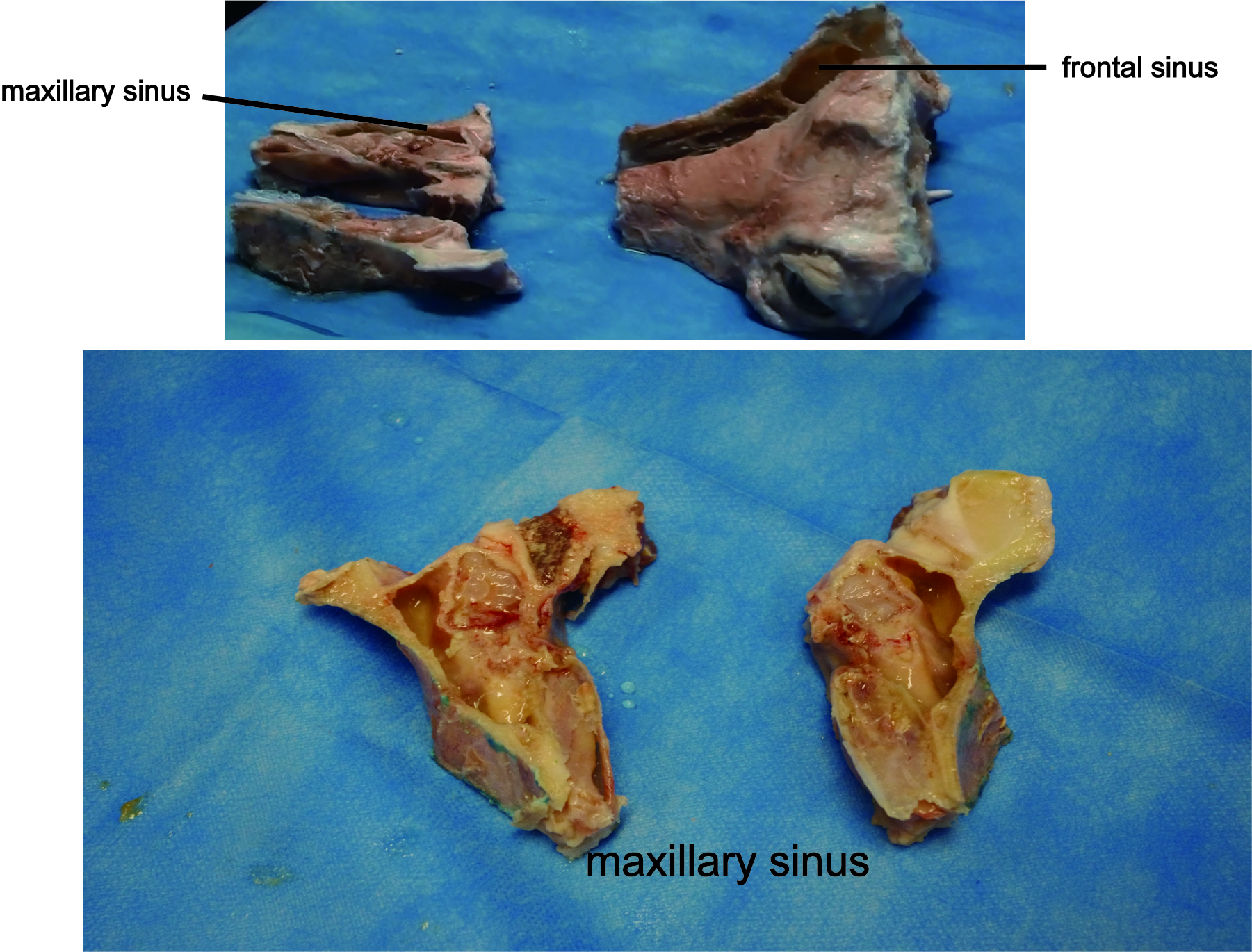

Supplement: Supplementary file 2 — Additional file2: Fig.S2 A schematic drawing for maxillary sinus (the picture above, left) and frontal sinus (the picture above, right). A sagittal plane for maxillary sinus (the picture below). [file 12903_2022_2233_MOESM2_ESM.tif]
